# Impacts of stormwater on coastal ecosystems: the need to match the scales of management objectives and solutions

**DOI:** 10.1098/rstb.2019.0460

**Published:** 2020-11-02

**Authors:** Phillip S. Levin, Emily R. Howe, James C. Robertson

**Affiliations:** 1 The Nature Conservancy, Washington Field Office, 74 Wall Street, Seattle, WA 98195, USA; 2 School of Marine and Environmental Affairs, The University of Washington, Seattle, WA, USA

**Keywords:** stormwater, scale, operational objectives, killer whale, salmon

## Abstract

Despite their limited area relative to the global ocean, coastal zones—the regions where land meets the sea—play a disproportionately important role in generating ecosystem services. However, coastal ecosystems are under increasing pressure from human populations. In particular, urban stormwater is an increasingly important threat to the integrity of coastal systems. Urban catchments exhibit altered flow regimes that impact ecosystem processes and coastal foodwebs. In addition, urban stormwater contains complex and unpredictable mixtures of chemicals that result in a multitude of lethal and sublethal impacts on species in coastal systems. Along the western coast of the United States, we estimate that hundreds of billions of kilograms of suspended solids flow off land surfaces and enter the Northern California Current each year. However, 70% of this pollution could be addressed by treating only 1.35% of the land area. Determining how to prioritize treatment of stormwater in this region requires a clear articulation of objectives—spatial distribution of appropriate management actions is dependent on the life histories of species, and management schemes optimized for one species may not achieve desired objectives for other species. In particular, we highlight that the scale of stormwater interventions must match the ecological scale relevant to species targeted by management. In many cases, management and policy will require mechanisms in order to ensure that local actions scale-up to efficiently and effectively achieve management objectives. In the face of rapid urbanization of coastal zones, failure to consider the match of management and ecological scales will result in the continued decline of coastal ecosystems and the species they support.

This article is part of the theme issue ‘Integrative research perspectives on marine conservation’.

## 1. Introduction

Marine and coastal ecosystems are among the most diverse regions on Earth (e.g. [1,2]). Globally, they support the livelihoods of some three billion people [3], and provide a range of ecosystem services, including climate regulation, food security and coastal defence [4]. Even so, ocean biodiversity is jeopardized by an array of human activities [5–8], threatening the well-being of communities that depend on the ocean.

Numerous assessments of threats to ocean health have been conducted, and these analyses often highlight such pressures as fishing, habitat loss, shipping and climate change (e.g. [5,8–10]). Often, such marine-based threats are thought to have greater impacts on marine ecosystems than pressures occurring on land. For instance, Halpern *et al.* [10] found that the global impacts of fishing on marine ecosystems are about fourfold, and climate impacts are more than 50-fold, greater than that from land-based pollution. Given that the relative impact of land-based activities on the sea is low at a global scale (because its footprint is small compared with the scale of threats like climate change and fishing), conclusions regarding the global importance of large-scale threats is reasonable. Even so, such evaluations may underestimate the impact of spatially limited human activities if ecologically or culturally rich locations are strongly impacted. Interestingly, the public perception that land-based pollution is the most significant threat to marine ecosystems [11] may highlight a blind spot in existing quantitative global assessments (cf. [12]). Indeed, human-driven impacts often interact across multiple scales, with localized perturbations coupling with global-scale threats to greatly affect the stability, resilience and productivity of marine ecosystems [13].

Despite their limited area relative to the global ocean, coastal zones—the regions where land meets the sea—play a disproportionately importants role in generating ecosystem services [14,15]. Ecosystem services such as food provisioning, nutrient regulation, waste treatment processing, coastal protection, recreational opportunities, spiritual fulfilment and cultural identity are generated at much greater levels in coastal zones relative to other regions of the ocean [16,17]. For example, coastal zones support a wide range of fishing fleets that support millions of households and livelihoods and can drive economies over a range of scales [18].

However, coastal regions are also under increasing pressure from a growing human population. Nearly 37% of the global population resides in coastal regions that collectively constitute less than 6% of the Earth's total area [19]. Moreover, populations in coastal zones are expected to increase at greater rates relative to inland areas, further intensifying this issue [20]. As human populations grow, so too will impacts on coastal ecosystems. Numerous studies have documented that development and urbanization of marine coastal zones result in impacts that cross terrestrial, freshwater and marine realms, resulting in damage to ecosystem services provided by these regions [13,21–25].

In addition to crossing terrestrial, freshwater and marine ecosystems, urban impacts also cross multiple spatial scales. Localized urban conditions may acutely impact individual marine organisms living along urbanized shorelines where habitat loss and pollutants are concentrated [26]. Additionally, marine waters distant from shorelines are also affected by urban areas—the outflow of pollutants emanating from urban areas can be hundreds of kilometres in the ocean [23]. Because the impacts of urbanization transcend scale, it is important to distinguish between pressures that are intrinsic and extrinsic. Intrinsic pressures arise within a realm, with impacts largely constrained to that same domain. Extrinsic pressures arise within one realm, but produce impacts in adjacent systems. Intrinsic pressures are more easily managed and regulated, whereas extrinsic pressures are notoriously difficult to govern [16]. However, the sustainability of coastal ecosystems depends, in part, on effectively diminishing impacts of extrinsic land-based human pressures on coastal ecosystems.

## 2. Urban stormwater—a key extrinsic pressure on coastal ecosystems

One of the primary extrinsic impacts of urbanization on coastal ecosystems is urban stormwater runoff—the fastest-growing cause of surface water impairment in the United States [27]. Urbanization of forested and other natural landscapes creates impervious surfaces that alter the quality, quantity and routing of surface water runoff as it moves across the landscape during and after rain events. Urban stormwater is generally delivered directly to stream and river networks by drainage pipes and open ditches that follow road systems. These forms of human-constructed (i.e. grey) stormwater infrastructure efficiently convey stormwater runoff and associated pollutants to downstream drainage systems, thus effectively avoiding urban flooding. However, grey infrastructure systems often bypass wastewater management systems as well as natural filtration through soils, wetlands and other forms of vegetation [28], and this leads to significant adverse effects downstream [29,30]. In particular, urbanized watersheds suffer from ‘urban syndrome’—a condition that results in low abundance and survival of sensitive aquatic and coastal species [31,32].

## 3. Water quality versus water quantity stormwater impacts

Urban stormwater runoff impacts the quantity and quality of water, both of which adversely impact the ecological integrity of receiving waterbodies [32–35]. Disentangling the impact of water quality versus water quantity on species and ecosystem processes remains a challenging and active area of research [36].

### (a) Water quantity

Urban catchments display hydrologic flow regimes that are altered in magnitude, frequency, duration and timing compared with natural systems [36]. This hydrologic alteration occurs because impervious surfaces and piped drainage systems deliver surface runoff to nearby streams and rivers more efficiently than natural landcover conditions. The inability to absorb rain events, coupled with more efficient routing of water, results in degraded hydrologic flow regimes in urban systems with as little as 5–10% impervious surface area [33]. Because river flows have shaped the evolution of life-history strategies, many aquatic or aquatic-dependent species respond negatively to physical habitat changes associated with urban hydrologic regimes [36,37]. In particular, population declines of diadromous species, whose life histories occupy both fresh and marine systems, can relay the degradation of freshwater ecosystems to the marine environment as population shifts alter biological interactions and energy transfers [38]. Shifts in flow regimes also influence near-shore marine ecosystem processes such as nutrient flux, organic matter processing and ecosystem metabolism. Coastal foodwebs are inextricably linked to river ecosystems through the transport of organisms, nutrients and materials [39,40]; however, the ability to quantitatively connect changes in freshwater flow regimes to coastal marine ecosystems is not well developed.

### (b) Water quality

Owing to increased nutrient and contaminant loads carried by stormwater to nearby rivers, lakes and estuaries, urban stormwater has emerged as an imminent threat to coastal systems [31]. As such, urban hydrologists and ecotoxicologists are increasingly focusing on the impact of urbanization on water quality and aquatic species [31,41]. Urban stormwater runoff contains complex and unpredictable mixtures of chemicals [31]; however, heavy metals and hydrocarbons from motor vehicles and commercial land use, as well as pesticides and pharmaceuticals, are ubiquitous in urban catchments. In some cases, exposure to urban stormwater results in acute lethal effects. For example, adult coho salmon (*Onchorhynchus kisutch*) returning to urban creeks experience much higher mortality rates prior to spawning (greater than 50%) compared with coho returning to non-urban creeks (less than 1%) [42]. Experimental results point to compounds found in tyre-wear particle leachates as the likely cause of this pre-spawn mortality [43]. Indeed, coho pre-spawn mortality is correlated with a suite of conditions associated with urbanization, including road density, traffic intensity and degree of imperviousness in the watershed [34]. High rates of pre-spawn mortality have significant impacts to the long-term viability of coho populations [44], with far-reaching ramifications for both freshwater and marine foodwebs [45].

While acute impacts of stormwater like salmon pre-spawn mortality are dramatic, and have captured the attention of the public (e.g. [46]), most exposure to stormwater results in sublethal impacts [47]. In individual organisms, stormwater can alter physiology, resulting in such phenomena as pericardial oedema and sensory deprivation in juvenile fishes [31]. In turn, the physiological alteration can reduce survival or reproductive output or shift behaviour, and this can have long-term, multi-generational consequences [47]. Such effects on individuals can propagate to population- and community-level dynamics [47]. These impacts can then lead to impacts on ecosystem-level processes, such as nutrient cycling, carbon sequestration, water quality and ecosystem resilience [47]. For example, the collapse of Japanese smelt has been linked to a chain of sublethal foodweb impacts generated by the land-based application of neonicotinoids [48]. Importantly, the temporal and spatial scale of sublethal impacts of stormwater can be extensive. Some organic compounds (e.g. polychlorinated biphenyls (PCBs), DDT) persist in the environment and bioaccumulate in animal tissues for long periods of time and can be transferred over extensive spatial scales by highly migratory species [49,50].

## 4. Confronting stormwater threats to coastal ecosystems: a case study

Confronting land-based threats such as urban stormwater to marine ecosystems requires dealing with two overarching challenges: (i) the spatial separation between where a threat arises on the landscape and where the impact of that threat is realized; and (ii) the mismatch in the spatial scale of impacts versus the scale of governance. Using the Puget Sound region in the United States as a case study, we highlight the scale and magnitude of the urban stormwater problem, the implications for management and recovery of imperilled species, and finally highlight solutions for large-scale extrinsic stressors impacting marine diversity.

### (a) The magnitude of the stormwater problem

**Figure 1.**
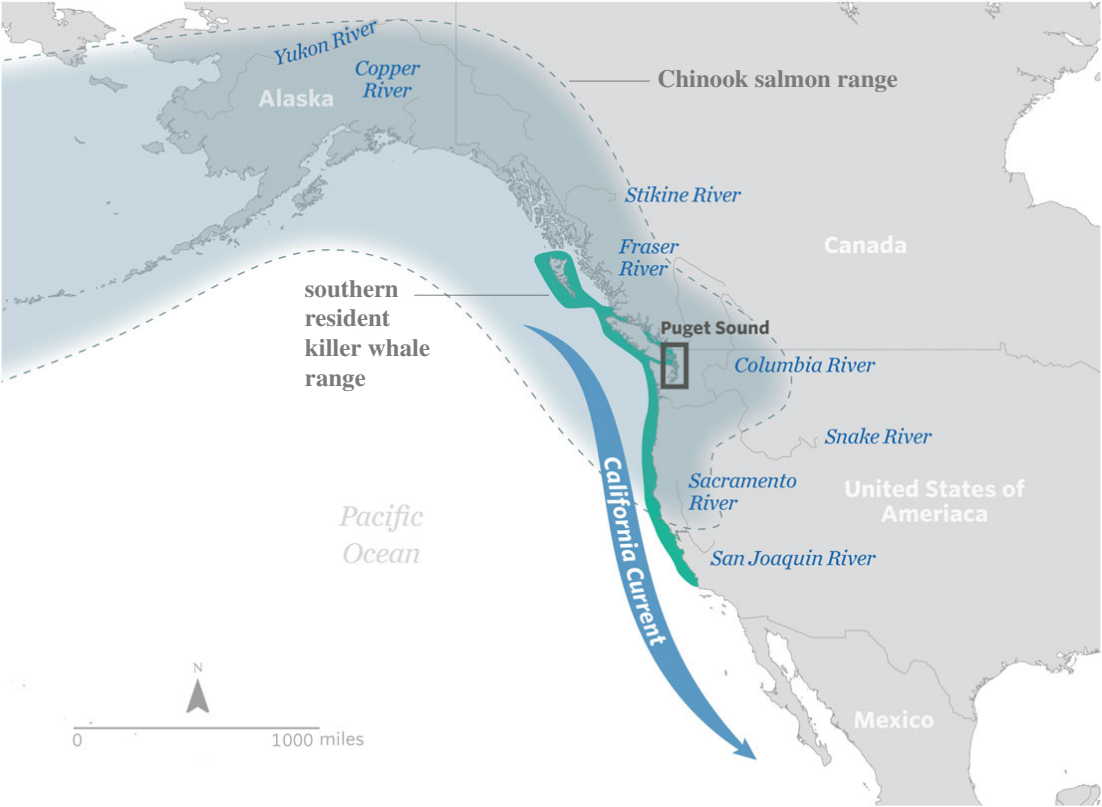
Puget Sound resides within the California Current Large Marine Ecosystem. The range of southern resident killer whales (teal shading) overlaps with the northern portion of the California Current. The range of Chinook salmon (blue shading) also includes the northern portion of the California Current.

**Figure 2.**
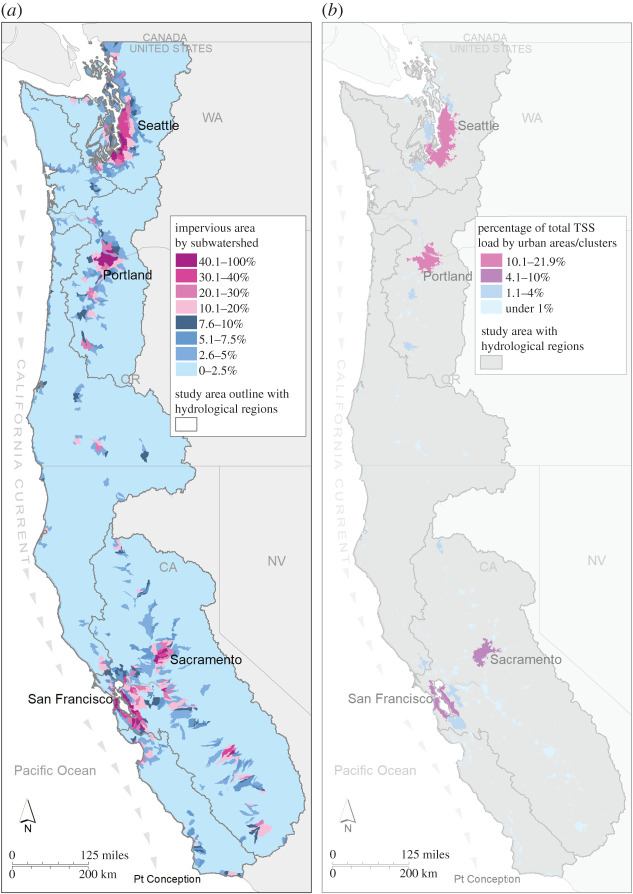
The US portion of the Northern California Current (from Point Conception, California to Canada) showing (*a*) the per cent cover of impervious area by subwatershed and (*b*) the loading of total suspended solids (TSS) by urban areas/urban clusters, expressed as the per cent of the total. Details are provided in the text and electronic supplementary material.

**Figure 3.**
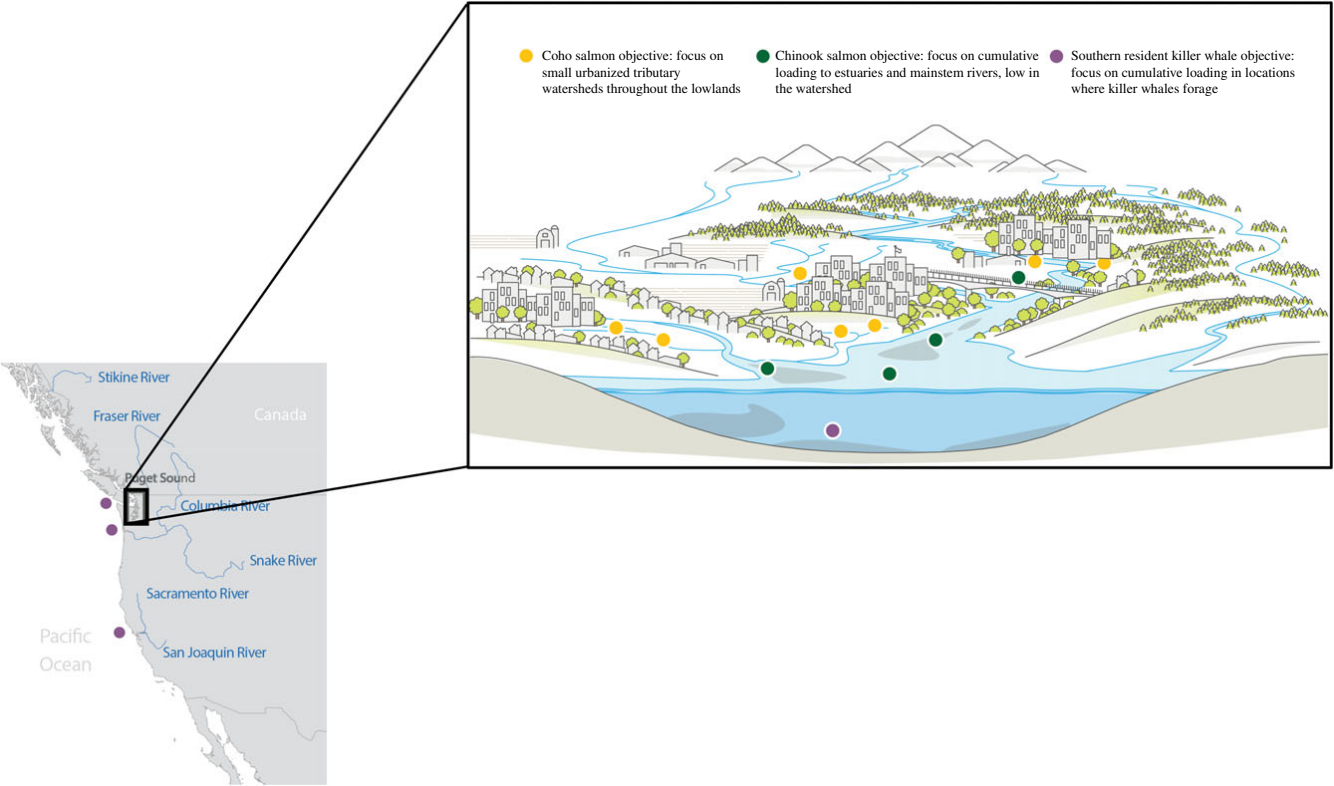
Caricature of the Puget Sound region. Yellow dots highlight the small, lowland tributaries where stormwater treatment would be most effective for coho salmon; green dots highlight the need for the cumulative reduction of stormwater contaminants in lower portions of mainstem rivers and estuaries in order to achieve operational objectives related to Chinook salmon; purple dots indicate the need to focus on a reduction of the loading in the Northern California Current resulting from cumulative inputs from urban areas.

Situated within the California Current Large Marine Ecosystem, the Puget Sound region encompasses 41 500 km^2^ of upland, freshwater, estuarine and marine habitats, and currently supports a large and increasingly urban population from Vancouver, British Columbia to Olympia, Washington (figures 1–3). Population projections suggest that human numbers in the greater Puget Sound region will increase by two million in the next 30 years [51]. With over 40 species of birds, mammals, fishes, plants and invertebrates currently listed as threatened, endangered, or candidates for state and federal endangered species lists, Puget Sound is considered a ‘hot-spot’ of extinction risk [52]. Importantly, some of these imperilled species, such as Chinook salmon (*Onchorhynchus tshawytscha*) and killer whales (*Orcinus orca*), are regional icons that have been commemorated in art, culture and tradition for millennia (e.g. [47]).

In the Pacific Northwest, the iconic nature of salmon and killer whales leads many stormwater programmes to highlight salmon recovery and ‘healthy’ killer whale populations as a goal (e.g. [51]). In Washington State, the local population of killer whales (southern resident killer whales, SRKW) are considered endangered under the Endangered Species Act, and specifically identified as an ecological endpoint in stormwater management plans. Tackling the stormwater problem for these species requires that we first determine the magnitude of the stormwater problem and how it is distributed across the habitat range of salmon and killer whales.

To assess the magnitude and distribution of stormwater impacts to salmon and killer whales, we calculated a pollution load metric by coupling annual hydrology data with regional pollution loading coefficients for different land use types. We focused on one pollution metric, total suspended solids (TSS), which is a commonly used metric to generally assess water quality, and includes a wide variety of materials that can be trapped on a filter, such as silt, decaying plant and animal matter, industrial waste, vehicle exhaust emissions, pavement wear, vehicle parts and oils, building materials and paints, and atmospheric particle deposition. Importantly, TSS does not fully capture water quality issues, as it does not account for dissolved pollutants; however, TSS is a useful indicator for urban stormwater pollution [53–56]. For this case study, we bounded our analysis to the northern portion of the California Current Ecosystem along the Pacific coast of the United States (hereafter referred to as the Northern California Current), which encompasses most of the home range of the SRKW and key portions of the Chinook salmon range (figure 1). We used a well-established stormwater pollution model [57] to calculate a TSS load for United States Geological Survey (USGS) watersheds (10-digit Hydrologic Unit Codes) at a 30 m spatial resolution and with regionally relevant TSS concentrations for six land use types [58–60]. Our intent is to roughly illustrate the magnitude and breadth of the stormwater pollution issue in the study area, and we acknowledge that there may be broad statistical error associated with the approach arising from spatial data input accuracy, the variability of the TSS concentrations and simplicity of the load algorithm. Additional methodological details are provided in the electronic supplementary material.

We calculated that 2.2282 × 10^11^ kg TSS flows from urban areas in watersheds associated with the Northern California Current system each year. This is a 1099% increase in TSS loads relative to pre-development conditions without impervious surfaces. Pre-development landscapes generated an average annual TSS export load of 40 kg per hectare [61]. Developed surfaces generate considerably more TSS, with freeways generating the highest loads (635 kg per hectare), followed by commercial surfaces (251 kg per hectare), industrial zones (219 kg per hectare), mixed residential (126 kg per hectare) and residential uses (86 kg per hectare) [61].

While hundreds of billions of kilograms of TSS are delivered each year to coastal marine systems in the Northern California Current, the generation of stormwater pollution is spatially heterogenous across the landscape and concentrated in urban areas. Urban areas hold 89% of the total human population in the region, and they generate 88% of the total TSS load. The watersheds feeding into San Francisco Bay contribute the largest amount of stormwater pollution (37% of the total TSS load) to the Northern California Current system, followed by the lower Columbia River watersheds (19%) and the Puget Sound watershed (15%) (table 1). Combined, these three basins generate 70% of the total TSS load in the Northern California Current. If we narrow our analysis to only pollution generated in urbanized areas of at least 10 000 people (US Census Bureau delineation of urban versus rural [62]) adjacent to the Northern California Current, 51% of the total TSS load is generated in just four cities: Seattle (22%), Portland (16%), San Francisco (8%) and Sacramento (5%). Because the generation of stormwater is concentrated in these four urban areas which occupy a small proportion of the total land area of the region, addressing 70% of the stormwater pollutant loading in the Northern California Current Ecosystem requires treatment of only 1.35% of the land area (table 1).

**Table 1.** Impervious area, total suspended solids (TSS load) and human populations are concentrated in three major watershed systems in the Northern California Current ecosystem. SF, San Francisco.

	SF Bay watershed (%)	lower Columbia watershed (%)	Puget Sound watershed (%)	all other coastal watersheds (%)
per cent impervious of entire study area^a^	0.69	0.31	0.35	0.59
per cent impervious of watershed	1.43	2.35	3.59	2.05
per cent of total impervious area	36	16	18	30
per cent of total TSS load	37	19	15	30
per cent of total human population	33	15	20	32

^a^For example, 0.69% of the total study area is developed impervious area of the SF Bay watershed.

### (b) Solving the stormwater problem requires clear operational management objectives

Solving the stormwater problem forces us to ask—solve for what? In order to effectively and efficiently prioritize natural resource interventions, clear objectives that translate high-level policy statements into action are required [63,64]. Objectives are commonly divided into two types—strategic and operational. Strategic objectives unpack high-level statements into declarations of what is to be achieved, and are focused on particular social, ecological, institutional or economic elements in a social–ecological system [65]. Operational objectives are derived from strategic objectives and are specific, measurable, achievable, realistic and time-bound [66]. Developing effective portfolios of operational objectives requires (i) a clear articulation of what management actions will and will not do [67], and (ii) that operational objectives for each major endpoint of the social–ecological systems—ecological, economic, social/cultural and institutional—be considered [68,69].

Here, we highlight how the location and scale of required stormwater interventions vary with the operational objective of management. We focus on three species—coho salmon, Chinook salmon and SRKW —to illustrate how the spatial distribution of appropriate management actions is dependent on the disparate life histories of these species, and how management schemes optimized for one species may not achieve desired objectives for other species.

#### (i) Stormwater mitigation for coho salmon

After spending 1–3 years in the ocean, coho salmon return to freshwater to spawn and rear for 1–2 years in small tributary creeks throughout Puget Sound lowlands [70,71] As described above, coho are acutely sensitive to toxic stormwater runoff when they return from the ocean to their natal creeks to spawn. They are also sensitive as juveniles, showing sublethal developmental impairments, such as pericardial oedema [31]. In both cases, the adverse effects of stormwater on coho occur while fish occupy their preferred freshwater habitats, small lowland creeks [72], rather than over a lifetime of contaminant accumulation. Stormwater interventions intended to improve the status of coho populations must focus on locations where coho spawn and rear. Thus, interventions concentrated on small tributary creeks in locations where stormwater composes the majority of creek flow will be most effective. Concentrating stormwater interventions to reduce overall pollution in the lower mainstems of river basins and marine waters, such as Puget Sound or San Francisco Bay, will pull resources towards the largest sources of pollution generation (e.g. industrial and commercial centres of large urban areas) rather than the small freshwater spawning habitats critical for coho salmon. Thus, mitigation of stormwater impacts on coho salmon requires a localized strategy that addresses contaminant loading in the specific creeks where coho rear and spawn.

#### (ii) Stormwater mitigation for Chinook salmon

By contrast to coho salmon, the impacts of stormwater pollution on Chinook salmon are more complex. In Puget Sound, Chinook salmon spawn primarily in the relatively non-urbanized upper reaches of major river basins and thus do not suffer from acute pre-spawn mortality as observed in coho [73]. However, stormwater toxics appear to have numerous impacts on Chinook in other portions of their life cycle. For example, juvenile Chinook that pass through estuaries impacted by stormwater pollution exhibit a 45% reduction in survival during their ocean residence relative to fish that migrate through uncontaminated habitats [74]. The mechanisms causing increased mortality in Chinook are ostensibly multifaceted, but reduced pathogen resistance and susceptibility to infection appear to be important (e.g. [58–60]). Thus, for species like Chinook that spawn and rear distant from urban centres, but must pass through contaminated estuaries, it is the cumulative loading of toxic compounds in the watershed that is critical. Management strategies that effectively and efficiently reduce the magnitude of toxics reaching estuaries should be favoured. For example, interventions that target lower reaches of large tributaries will be more efficient in reducing large quantities of pollution compared with actions in small streams in the upper reaches of the watershed.

#### (iii) Stormwater mitigation for southern resident killer whales

SRKW consist of three fish-eating pods of killer whales that range from Northern California to Southeast Alaska [75] (figure 1). In 2005, they were listed as endangered under the Endangered Species Act, and in the listing the National Marine Fisheries Service highlighted high levels of contaminants from stormwater was a key threat [75]. In particular, persistent organic pollutants (POPs) such as polychlorinated biphenyls and polybrominated diphenyls occur in SRKW. These chemicals are an important component of stormwater, and bioaccumulate through foodwebs, building up and persisting in the body tissues of organisms over time. As the concentration of these chemicals increases in their tissues, organisms experience adverse health effects including endocrine disruption, reproductive disruption, immunotoxicity, neurotoxicity, neurobehavioural disruption and cancer [76].

Adult killer whales are primarily exposed to POPs through the ingestion of prey. Given the dominance of Chinook salmon in the diet of killer whales [77], and the elevated POP levels in Chinook [76], these fish are the primary source of contaminants in SRKW. Chinook salmon that feed in Puget Sound have high contaminant concentrations, likely a result of both the proximity to urban areas and high residence time of water in Puget Sound [78,79]. Consequently, killer whales that spend a significant amount of time foraging in Puget Sound are exposed to high contaminant levels [80]. Even so, because Chinook acquire greater than 96% of their body burden of POPs from marine habitats [79,81], and SRKW forage across the Northern California Current [82], feeding on salmon from a diversity of watersheds [83], whales will be exposed to pollution generated from urban areas across the region. This may be particularly relevant in years of low salmon abundance because even those whales known to primarily forage in Puget Sound will spend more time elsewhere in search of prey [84].

Because some whales reside in Puget Sound in summer months and feed on Chinook with high levels of contaminants, reducing the cumulative loading of toxic compounds in Puget Sound will certainly have some positive benefit. Indeed, the governor of Washington state has proposed spending $51 million to reduce and manage stormwater in Puget Sound in an effort to recover killer whales [85]. However, for SRKW that use habitats across the Northern California Current ecosystem [86], local efforts in one geographical location may be insufficient. In order to manage stormwater effectively for SRKW, efforts may be required across the entirety of their home range. As this includes 1621 municipalities, 82 counties and three states in the United States and additional jurisdictions in Canada, this is a formidable task. Nonetheless, the same approaches used to prioritize conservation actions in other highly migratory species (e.g. [72–74]) could be adapted to develop effective and efficient stormwater mitigation at a scale relevant to killer whale life history.

## 5. Policy solutions for effective stormwater management

The Puget Sound case study highlights a number of key issues that, if successfully addressed, could lead to more effective outcomes in stormwater water management. Here, we highlight three actions governance bodies could implement that would yield substantial benefits—development of scale-appropriate operational objectives, increased connectivity across scales of governance, and implementation of stormwater credit programmes.

Stormwater management is implemented at the operational level through management plans, administrative regulations and decisions of individual managers or institutions. Dozens of potential management actions have been developed and shown to be effective for reducing the toxicity of stormwater (full evaluation and cost-effectiveness of these actions can be found in [87]) For instance, sand filtration of stormwater through a pretreatment system, flow spreaders, a sand bed and underdrain piping can reduce TSS by 80% [88]. Street sweeping can also effectively remove pollutants from stormwater. In a study of street sweeping effectiveness in Korea, sweeping reduced event mean concentrations of TSS by 78% [89]. Green stormwater infrastructure mimics natural processes by encouraging stormwater to infiltrate into the ground by slowing down flows and filtering out pollutants. This type of treatment significantly reduces the quantity of stormwater and improves the quality of stormwater runoff. For instance, constructed wetlands can remove more than 70% of metal pollution in stormwater [88].

No matter what management tactic is employed, effective management is contingent on operational objectives that are linked to feasible and measurable indicators and reference levels [90]. In the United States, because stormwater management is typically implemented at the scale of cities and counties [28], operational objectives must target problems at scales that match the governance or the problems themselves, while policy instruments are required to overcome a scale mismatch between objectives and governance [91]. For example, in the Puget Sound region, operational objectives focused on local populations of coho salmon can provide a clear link between specific management actions and ecological outcomes. Operational objectives focused on Chinook salmon may require coordination among several municipalities spread across many watersheds that all contribute contaminants to mainstem rivers and estuaries. For SRKW, meeting specific operational objectives may require collaboration among the large urban areas across the Northern California Current. We suggest operational objectives created by local management bodies for issues that must be addressed at a larger scale are problematic unless a plan for coordination is in place for expanding conservation actions to the appropriate scale [92].

Effective governance depends on collaboration, social learning and integration of knowledge across actors. Thus, well-developed networks of local actors are crucial for successful management for issues that can be addressed at local scales, such as in coho salmon. Fortunately, governance and management of stormwater are characterized by a high concentration of managers and organizations that are often well integrated [93]; therefore, prospects for successful local-scale outcomes are high. However, networks among individuals or institutions that are active at different scales and could promote cross-scale linkages are often absent [94]. Networks that cross scales and link otherwise unconnected governance structures and actors are crucial for reaching objectives that require large-scale cooperation. Explicitly engaging scale-crossing individuals will be critical for successfully achieving goals associated with objectives that are inherently multi-scalar, such as those associated with killer whales and Chinook salmon [95]. Scale-crossing brokers [96] will not only improve coordination across scales, they can also create new pathways for exchanging information and incubating innovation [95]. Further, the ability to detect and act on gradual system changes requires knowledge derived in different places and at varying scales. Thus, scale-crossing brokers serve as an important component of adaptive management by blending and conveying critical monitoring information.

Another approach to encourage cross-network collaboration that has shown promising results for stormwater is the implementation of cap and trade policies targeting total daily maximum loads (TMDLs) at the catchment or watershed scale. These regulations encourage management performance and progress towards meeting operational objectives, as opposed to compliance with a set of construction guidelines [29]. Because TMDLs focus on performance, they enable integrated credit trading among polluters, which in turn encourages cross-sector collaboration. With the implementation of nutrient TMDLs in Chesapeake Bay, for example, agriculture, wastewater and stormwater departments and technologies began to work in concert with one another. The result, at the time of the midpoint assessment, was the highest estimates of water quality over a 30-year period [27].

While TMDLs achieve coordination through mandates, scale-crossing among organizations incentivized by cap and trade policies and associated funding may also achieve management at appropriate spatial scales. Mandated coordination alone is rarely viewed as productive [93]. Thus, a shared interest generated by credit trading policies with complementary funding may achieve success in some instances.

## 6. Conclusion

The phrase ‘think globally, act locally’ has become an iconic principle of the modern environmental movement. Typically, this maxim is meant to inspire and encourage individuals to perform local conservation actions with the assumption that these actions will coalesce to create a desirable global future [97]. However, when first articulated by Dubos [98], the phrase was meant to warn environmentalists that global objectives cannot easily be translated into local actions [97]. Such may be the case with stormwater. While local stormwater actions may be meant to solve large-scale issues confronting coastal ecosystems, they will often be inadequate by themselves. Local actions must be coordinated into systems of effective management that operate on spatial scales appropriate to specific management objectives. The informal and formal means to achieve such coordination are available, but it will take recognition by actors working at all scales that cross-scale collaboration is crucial before it will become normal and commonplace. Given the rapid urbanization of coastal zones and the concomitant increase in stormwater contamination, the integrity of coastal and marine ecosystems depends on us thinking globally while we act at all scales.

## Supplementary Material

Appendix A: Methological details

## Data accessibility

This article has no additional data.
