# Supplementary material for: Impacts of stormwater on coastal ecosystems: the need to match the scales of management objectives and solutions
Source: Philos Trans R Soc Lond B Biol Sci. 2020 Nov 2;375(1814):20190460. doi: 10.1098/rstb.2019.0460 (PMC7662197; doi:10.1098/rstb.2019.0460)
Supplement: Appendix A: Methological details [file rstb20190460supp1.docx]

Appendix A:

## Methods for calculating stormwater runoff and Total Suspended Solids (TSS) load

We conducted spatial data processing and analysis with Google Earth Engine (Gorelick et al. 2017). The study area consisted of watersheds flowing from the USA states of Washington, Oregon, and California to Pacific coastal waters south of the Canadian border to Point Conception, California. To roughly avoid complexities of large reservoirs and extreme transport distance, the analysis excludes areas of the Columbia Basin upstream of Bonneville Dam and areas upstream of the Shasta Dam on the Sacramento River. The analysis may significantly under-calculate Total Suspended Solids (TSS) loads by not including stormwater from those excluded areas. Likewise, rural areas were not well-accounted for which likely lead further under-calculation of total stormwater runoff and load. Conversely, some over-calculation of TSS load may also occur because we do not address fate and transport of TSS loads in our approach.

To calculate stormwater runoff and TSS load, we employed the Simple Method created by the Center for Watershed Protection (CWP 2003), which was based on Schueler (1987). For load, we applied median observed concentrations associated with specific land use types to account for differences in TSS concentrations present on various land use areas.

Annual Pollutant Load (from CWP 2003, p.61)

| The Simple Method estimates pollutant loads as the product of annual runoff volume and pollutant EMC, as:  **L = 0.226 * R * C * A**  Where: L = Annual load (lbs), and:  R = Annual runoff (inches)  C = Pollutant concentration in stormwater, EMC (mg/l)  A = Area (acres)  0.226 = Unit conversion factor |
| --- |

Annual Runoff (from CWP 2003, p.61)

| The Simple Method calculates the depth of annual runoff as a product of annual runoff volume and a runoff coefficient (Rv). Runoff volume is calculated as:  **R = P * Pj * Rv**  Where: R = Annual runoff (inches), and:  P = Annual rainfall (inches)  Pj = Fraction of annual rainfall events that produce runoff (usually 0.9)  Rv = Runoff coefficient  In the Simple Method, the runoff coefficient is calculated based on IC in the subwatershed. The following equation represents the best fit line for the data set (N=47, R^2^=0.71).  **Rv = 0.05 + 0.9Ia**  Where: Rv = runoff coefficient, and:  Ia = Impervious fraction |
| --- |

We calculated runoff and load for each 30-meter pixel (900 m^2^) within a northern and southern region of the study area. The area of each pixel was derived and applied to each pixel load on the fly as directed in our GEE code using pixel size of the National Land Cover Dataset (Yang et al. 2018) input. We split the two regions on boundaries of USGS Hydrologic Unit Code 4-digit polygons (USGS 2017) where they were near to the zonal boundaries of broadly recognized rainfall regimes. This division occurs just north of the San Francisco Bay area, so coastal watersheds north of and not flowing into San Francisco Bay are included in the northern region.

We calculated pre-development runoff and load by reassigning all development classes (Residential, Commercial, Industrial, and Roads) to the Open Space category. Change from pre-development to current was calculated as the difference between those two scenarios, and percent change as:

(current - predevelopment) / predevelopment

While this approach likely over-calculates runoff and load (because load concentrations in non-developed vegetated areas would be lower than in developed Open Space), it provided a rough, conservative result.

We performed runoff and load comparisons with two sets of analysis units. For runoff and loads of urban areas, we used the US Census Bureau (2016) Urban Areas and Clusters (UAC). For regional runoff and loads, we grouped HUC polygons into four units: those feeding the 1) Puget Sound, 2) Columbia River, 3) San Francisco Bay, and 4) all others, i.e. small coastal watersheds.

Input data included:

**Annual rainfall:** We used PRISM (Parameter-elevation Regressions on Independent Slopes Model) Long-Term Average Climate Dataset Norm81m for annual precipitation (Daly et al. 2008). This dataset was referenced in GEE code as “OREGONSTATE/PRISM/Norm81m” and by selecting band “ppt”.

**Impervious fraction:** We used the 30-meter resolution National Land Cover Dataset 2011 impervious surface layer, which provides the percent of each pixel that is human-made impervious surface (Yang et al. 2018). This dataset was referenced in GEE code as “USGS/NLCD/NLCD2011” and by selecting band “impervious”.

**Land use:** We used a land use dataset from Messager (2018) which was a combination of statewide land use/zoning datasets, the 30-meter resolution National Land Cover Dataset 2011 (NLCD) land cover layer, and US Census Tiger road lines dataset. The dataset consisted of five broad land use categories which were associated with median observed TSS concentrations.

| *Land Use Type* | *Input Data Source (NLCD values / zoning category)* |
| --- | --- |
| Waterbodies | NLCD Water (11) |
| Open Space | NLCD Developed, Open Space (21) |
| Residential | NLCD Developed, Low and Medium Intensity (22, 23) |
| Commercial | Zoning commercial or other; NLCD Developed High Intensity (24) |
| Industrial | Zoning industrial; NLCD Developed High Intensity (24); industrial zoning |
| Roads | NLCD Roads (96); A final step in developing this dataset was a reclassification to categorize a pixel as Road if for any developed pixel (21, 97, 98, 99), if 12-m buffered roads cover more than 50% of the impervious area in the developed pixel (NLCD values 21, 97, 98, 99), so (100*(rasterized roads)/(25*imperviousness) > 0.50). |

**TSS Concentration coefficients:** We applied region-specific median observed TSS concentrations to a northern and a southern region of the study area. For the northern region, we used concentration values provided by Geosyntec Consultants, Inc. (2018), which were derived primarily from two sources: Washington State Department of Ecology 2015 and Washington State Department of Transportation 2015. For the southern region, we used national level concentration values from “The National Stormwater Quality Database (NSQD)” (Pitt et al. 2018). The TSS concentrations by region for each land use type are:

| Land Use Type | TSS Concentration (g/L) | |
| --- | --- | --- |
|  | North Region^i^ | South Region^ii^ |
| Waterbodies | 0 | 0 |
| Open Space | 14 | 38 |
| Residential | 28 | 56 |
| Commercial | 41 | 52 |
| Industrial | 48 | 74 |
| Roads | 66 | 74 |

^i^ Geosyntec Consultants, Inc., 2018, produced primarily from two sources: Washington State Department of Ecology 2015; Washington State Department of Transportation 2015.

^ii^ Pitt et al. 2018, pp.23

Citations

Daly, C., Halbleib, M., Smith, J.I., Gibson, W.P., Doggett, M.K., Taylor, G.H., Curtis, J., and Pasteris, P.A. 2008. Physiographically-sensitive mapping of temperature and precipitation across the conterminous United States. *International Journal of Climatology*, 28: 2031-2064

Geosyntec Consultants, Inc. 2018. “Puget Sound Stormwater Decision Support Tool.” *In draft*.

Gorelick, N., Hancher, M., Dixon, M., Ilyushchenko, S., Thau, D., & Moore, R. 2017. Google Earth Engine: Planetary-scale geospatial analysis for everyone. *Remote Sensing of Environment*.

Messager, M. 2018. Unpublished digital data. Accessed on November 16, 2018. Methods described at https://github.com/messamat/PacificStormwater.

Pitt, R., Maestre, A., and Clary, J. 2018. “The National Stormwater Quality Database (NSQD), Version 4.02.” Accessed on January 1, 2020, at http://www.bmpdatabase.org/Docs/NSQD_ver_4_brief_Feb_18_2018.pdf

US Census Bureau. 2016. TIGER/Line Shapefile, 2016, 2010 nation, U.S., 2010 Census Urban Area National. Accessed on November 16, 2018, at http://www2.census.gov/geo/tiger/TIGER2016/UAC10/tl_2016_us_uac10.zip

USGS. 2017. HUC04: USGS Watershed Boundary Dataset of Basins. Accessed on November 16, 2018, at https://developers.google.com/earth-engine/datasets/catalog/USGS_WBD_2017_HUC04

Washington State Department of Ecology. 2015. “Western Washington NPDES Phase I Stormwater Permit Final S8. D Data Characterization.” https://fortress.wa.gov/ecy/publications/SummaryPages/1210050.html.

Washington State Department of Transportation. 2015. “WSDOT NPDES Municipal Stormwater Permit Final Highway Runoff Characterization Report (S7. B) Water Years 2012-2014.” October.

Yang, L., Jin, S., Danielson, P., Homer, C., Gass, L., Case, A., Costello, C., Dewitz, J., Fry, J., Funk, M., Grannemann, B., Rigge, M. and G. Xian. 2018, A New Generation of the United States National Land Cover Database: Requirements, Research Priorities, Design, and Implementation Strategies, p. 108–123.
